# A Novel FACS-Based Workflow for Simultaneous Assessment of RedOx Status, Cellular Phenotype, and Mitochondrial Genome Stability

**DOI:** 10.3390/biochem1010001

**Published:** 2021-04-02

**Authors:** Patrick M. McTernan, Paige S. Katz, Constance Porretta, David A. Welsh, Robert W. Siggins

**Affiliations:** 1 Comprehensive Alcohol Research Center, Louisiana State University Health Sciences Center, New Orleans, LA 70112, USA;; 2 Department of Physiology, Louisiana State University Health Sciences Center, New Orleans, LA 70112, USA;; 3 Department of Medicine, Section of Pulmonary and Critical Care Medicine, Louisiana State University Health Sciences Center, New Orleans, LA 70112, USA

**Keywords:** FACS, RedOx status, mitochondria, mitohormesis, mtDNA

## Abstract

Intracellular reduction-oxidation (RedOx) status mediates a myriad of critical biological processes. Importantly, RedOx status regulates the differentiation of hematopoietic stem and progenitor cells (HSPCs), mesenchymal stromal cells (MSCs) and maturation of CD8+ T Lymphocytes. In most cells, mitochondria are the greatest contributors of intracellular reactive oxygen species (ROS). Excess ROS leads to mitochondrial DNA (mtDNA) damage and protein depletion. We have developed a fluorescence-activated cell sorting (FACS)-based protocol to simultaneously analyze RedOx status and mtDNA integrity. This simultaneous analysis includes measurements of ROS (reduced glutathione (GSH)), ATP5H (nuclear encoded protein), MTCO1 (mitochondrial DNA encoded protein), and cell surface markers to allow discrimination of different cell populations. Using the ratio of MTCO1 to ATP5H median fluorescence intensity (MFI), we can gain an understanding of mtDNA genomic stability, since MTCO1 levels are decreased when mtDNA becomes significantly damaged. Furthermore, this workflow can be optimized for sorting cells, using any of the above parameters, allowing for downstream quantification of mtDNA genome copies/nucleus by quantitative PCR (qPCR). This unique methodology can be used to enhance analyses of the impacts of pharmacological interventions, as well as physiological and pathophysiological processes on RedOx status along with mitochondrial dynamics in most cell types.

## 1. Introduction

Cellular reduction-oxidation (RedOx) status is a crucial determinant of multiple biological processes, including the regulation of embryonic and adult stem cell self-renewal and differentiation [1]. Adult stem cells are rare in most organs and tissues, necessitating methods to examine RedOx status on a single cell level. Several in vivo and in vitro methods exist to examine the RedOx state in tissues and cells. However, these protocols do not measure multiple parameters of RedOx status (antioxidant analysis) and give an indication of mitochondrial (mt)DNA integrity in phenotypically defined cells [1–3].

Fluorescence-activated cell sorting (FACS) permits both surface and intracellular phenotyping and sorting for a myriad of downstream analyses, including transcriptional and DNA profiling. To determine oxidative burden, reducing capacity, and mtDNA integrity, we developed a methodology using a reactive oxygen species (ROS) indicator dye, followed by immunostaining for markers of specific cell populations. This staining protocol included CD105 (mesenchymal stromal cell (MSC) marker), CD34 (hematopoietic stem/progenitor cell (HSPC) marker), CD3, CD4, and CD8 (T cells), total reduced glutathione (GSH), MTCO1 (mtDNA-encoded protein), and ATP5H (nuclear DNA-encoded protein). Higher ratios of MTCO1 to ATP5H indicate “healthier” mitochondria [4].

Unlike previously described protocols, this workflow allows for simultaneous measurements of both RedOx status (by measuring reduced glutathione and reactive oxygen species) and mtDNA integrity (indicator of genomic stability) [5–13]. Most protocols for measuring ROS require living cells and specific staining buffers that only allow for parallel measurements of other factors, such as mitochondrial health [6]. While these data are reliable, parallel measurements preclude analysis of both parameters within a single cell. Additionally, fluorescence cell barcoding allows multiple treatment groups or samples to be immune-stained simultaneously, ensuring less variability in results and conserving reagents [14]. Finally, fixed cells can be sorted at a BSL-2 containment level for downstream assays, such as qPCR for mtDNA quantification, whereas unfixed live human subject samples harboring infectious agents require a BSL-3 level containment [15].

To validate this method, we utilized three different cell types. First, we treated MUTZ-3 cells (model of CD34+ HSPCs) with pyocyanin (H_2_O_2_ generator) [16]. As expected, the treated cultures showed increased oxidative burden and a higher level of depolarized mitochondria. We also found that ROS production positively correlated with mitochondrial polarization. Second, we used human PBMCs, and observed a dose-dependent increase in MTCO1:ATP5H ratio, with increasing doses of ethidium bromide in CD8+ T cells. Analysis of expression of transcripts that regulate ReDox state and mitochondrial biogenesis revealed expected increases in expression of antioxidant genes, as well as genes that regulate mitochondrial biogenesis. Third, we performed ex vivo studies on MSCs isolated from chronic binge alcohol (CBA)-administered or control rhesus macaques [17]. MSCs isolated from CBA-administered macaques had higher levels of both ROS and GSH than control MSCs, though the ratio of ROS:GSH, an index of oxidative burden, is not altered between these groups. Further stress with 50 mM in vitro alcohol resulted in increased ROS:GSH only in the MSCs isolated from CBA animals. Furthermore, the MTCO1:ATP5H ratio positively correlated with mtDNA quantity. These data suggest a mitohormetic response in CBA-MSCs without in vitro alcohol treatment, and that further oxidative stress leads to allostatic overload [18].

This workflow provides informative data on cellular hormetic and mitohormetic responses to oxidative stressors while utilizing only five fluorophores, leaving further channels to be utilized for cell phenotyping and/or signal transduction analysis. Finally, unlike many methods for FACS detection of ROS, we use a fixable, permeabilizable ROS-indicator dye that allows for intracellular antibody staining to identify and semi-quantify intracellular proteins for pathway analyses. To that end, we developed and optimized a FACS-based protocol to simultaneously analyze RedOx status (by analyzing the antioxidant glutathione and ROS) and mtDNA integrity (by measuring MTCO1 and ATP5H) that allows for downstream analyses on specific sorted cell populations.

## 2. Materials and Methods

### 2.1. MUTZ-3 Cell Culture and CD34+ Immunomagnetic Separation

MUTZ-3 cells (DSMZ, Brunswick, Germany) are a human acute myelomonocytic leukemia cell line containing multipotent hematopoietic progenitor cells (HPCs; CD34+) that are capable of differentiating into multiple myeloid lineages. Cells were maintained in RPMI 1640 containing (2% pen/strep, and 2 mM L-glu, 20% h.i. FBS, 20% conditioned media harvested from HTB-9 urinary bladder carcinoma cell line; (ATCC, Manassas, VA, USA) culture (RPMI-1640 + 10% h.i.FBS, 2% pen/strep, and 2 mM L-glu; Complete Conditioned Media (CCM)). Every 2 weeks, cells were immunomagnetically sorted using the human CD34+ cell isolation kit (Miltenyi, San Diego, CA, USA) according to manufacturer’s protocol. Acute in vitro oxidative stress was induced by a single 10 μM dose of pyocyanin (Enzo, Farmingdale, NY, USA) for 16 h. Long-term in vitro oxidative stress was induced with 2.5 μM pyocyanin every 3 days for 12 days.

### 2.2. Peripheral Blood Mononuclear Cell Culture

Cryopreserved peripheral blood mononuclear cells (PBMCs, Zen-Bio, Research Triangle Park, NC, USA, donors 1614B and 1614C; and Astarte Biologics, Bothell, WA, USA, donors 213 and 910) were quickly thawed at 37 °C while shaking gently. Cells were added slowly to 5 mL of pre-warmed OpTimizer T-Cell Expansion SF media (ThermoFisher Scientific, Waltham, MA, USA) plus IL-2 (30 U/mL, GoldBio, St. Louis, MO, USA), plus glutamine, Pen/Strep. Warm media was added to achieve 10 mL total volume and invert gently. Cells were centrifuge at 500*× g* for 5 min at room temperature (RT). Tubes were decanted, cell pellets were resuspended in 1 mL of warm media for cell enumeration, and cell density adjusted to 1 *×* 10^6^ cells/mL. The following day, cells were centrifuged at 500 *g* for 5 min at room temperature (RT), tubes decanted, and cell pellets resuspended in 1 Ml of warm media for cell enumeration. Cell density was again adjusted to 1 *×* 10^6^ cells/mL to account for overnight cell death. The previous steps were repeated on the following day and DynaBeads Human T-Activator CD3/CD28 (ThermoFisher Scientific, Waltham, MA, USA) added at 1:1, bead:cell ratio. Cell density was maintained at 1 × 10^6^ cells/mL. On the fifth day of culture, cell density was reduced to 5 *×* 10^5^ cells/mL. Fresh warm media was added when cell density reached 2 *×* 10^6^ cells/mL to reduce density to 5 *×* 10^5^ cells/mL during expansion. To deplete mitochondrial DNA (mtDNA), cells were treated with 0, 400, or 1200 ng/mL of UltraPure^™^ Ethidium Bromide (EtBr, ThermoFisher Scientific, Waltham, MA, USA) daily for 3 days, and cells harvested for analysis on day 4. Positive control cells were treated with IL-15 (10 IU/mL, GoldBio, St. Louis, MO, USA) [19]. IL-15 is a critical cytokine for regulating mitochondrial spare respiratory capacity (SRC) and oxidative metabolism by promoting mitochondrial biogenesis in T cells.

### 2.3. FACS Assay Reagents and Antibody Labeling

To maximize versatility of this FACS assay, non-species-specific dyes and unconjugated antibodies with a wide range of species cross-reactivity were chosen. CellTrace^™^ Violet Cell Proliferation Kit (ThermoFisher Scientific, Waltham, MA, USA) was used in PBMC experiments to barcode treatment groups [14]. Viability was assessed using Fixable Viability Dye eFluor^®^ 780 (ThermoFisher Scientific, Waltham, MA, USA). CellROX^®^ Green Reagent (Life Technologies, Grand Island, NY, USA) is a commercially available ROSsensitive fixable, permeabilizable dye. In the current studies, we used Anti-ATP5H (Abcam, Cambridge, MA, USA; clone 7F9BG1) conjugated with Lightning-Link PE-Cy7 Antibody Labeling Kit (Novus). Anti-MTCO1 (Abcam, Cambridge, MA, USA; clone 1D6E1A8) conjugated with SiteClick^™^ Qdot^®^ 655 Antibody Labeling Kit (Life Technologies, Grand Island, NY, USA) for MUTZ3 and PBMC experiments or Pacific Blue^™^ Antibody Labeling Kit (Life Technologies, Grand Island, NY, USA) for MSC experiments. Anti-Glutathione (Millipore, Billerica, MA, USA; 8.1GSH) was conjugated to APC (Lightning-Link APC Antibody Labeling Kit, Novus, Littleton, CO, USA). All antibody labeling reactions were performed according to the protocols supplied by the manufacturers. The HSPC panel consisted of the above reagents and anti-CD34-PE (BD Biosciences, San Jose, CA, USA; clone 563). In place of anti-CD34, the T cell Subset Panel contained anti-CD3-PerCP (BioLegend, San Diego, CA, USA; clone OKT3), anti-CD4-PE (BioLegend, San Diego, CA, USA; clone OKT4), and anti-CD8-AF700 (BioLegend, San Diego, CA, USA; clone RPA-T8). Anti-CD105-PE (eBiosciences, San Diego, CA, USA; clone SN6) was used to determine MSC phenotype.

### 2.4. Fluorescence Activated Cell Sorting (FACS) and Analysis of Cell Phenotype, RedOx Parameters, and Mitochondrial Genome Stability

**Figure 1.**
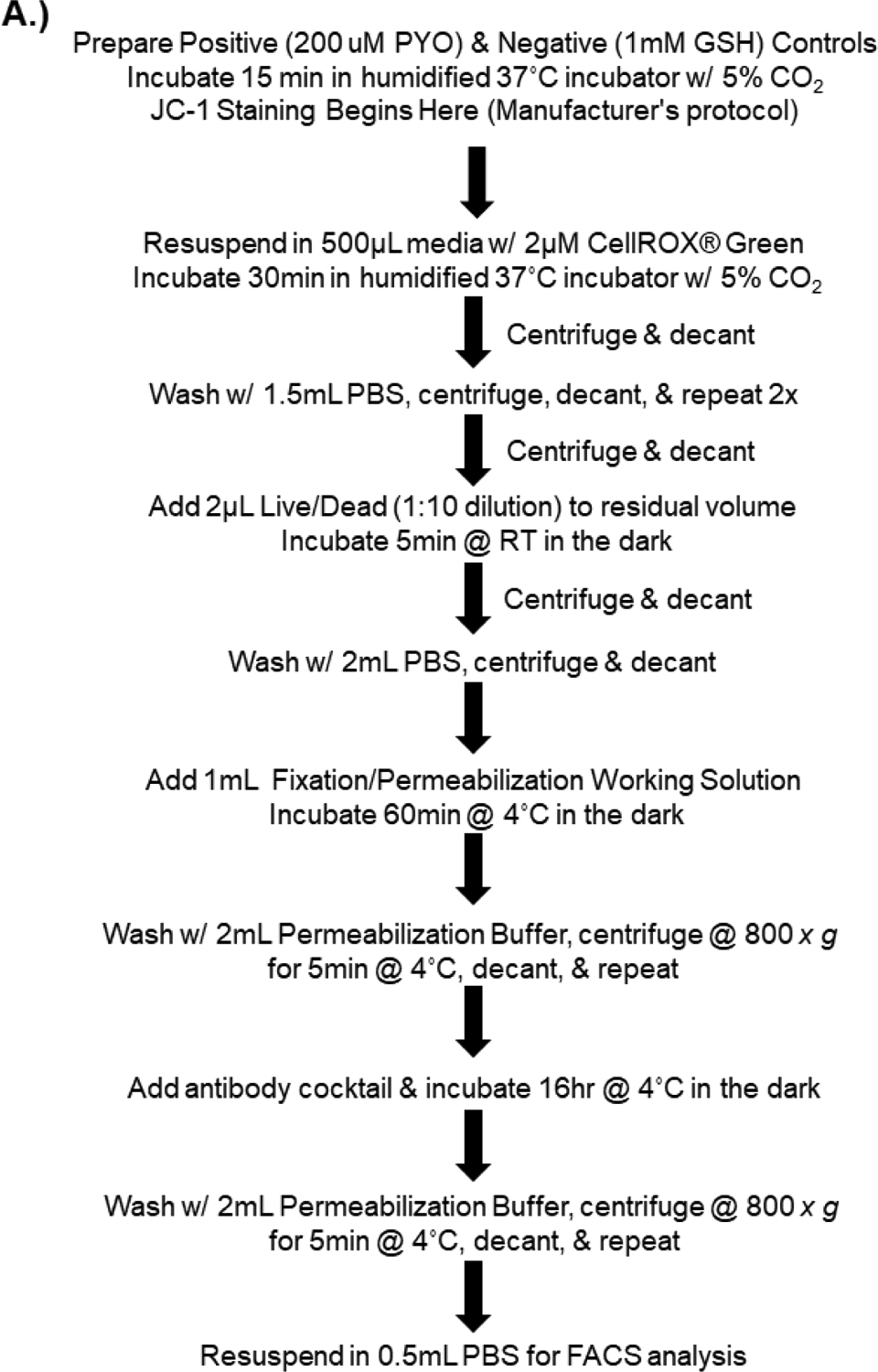
A.) Workflow for assessment of phenotype, mitochondrial health, and RedOx status. All centrifuge steps are performed at 500 *x g* for 5 min at 20 °C, followed by decanting unless stated otherwise.

For PBMC experiments, the 4 treatment groups were first barcoded according to EtBr treatment using CellTrace^™^ Violet Cell Proliferation Kit (ThermoFisher Scientific, Waltham, MA, USA) as follows: Control (0.04 μM), 400 ng/mL EtBr (0.16 μM), 1200 ng/mL EtBr (0.63 μM) and IL-15 (2.5 μM). After barcoding, all treatment groups for each donor were combined. The following remaining workflow is summarized in Figure 1. Positive and negative controls were prepared for the CellROX^®^ Green Reagent (Life Technologies, Grand Island, NY, USA) according to the manufacturer’s protocol with minor modifications. Pyocycanin (200 μM) was added to the positive ROS control, and N-acetyl-L-cysteine (1 mM, Sigma-Aldrich, St. Louis, MO, USA) was added to the negative ROS control (15 min for each treatment). Following incubation, cells were centrifuged at 500 *× g* for 5 min at 20 °C. After decanting, cells were resuspended in CCM without serum, and CellROX^®^ Green Reagent was added to achieve a final concentration of 2 μM. Samples were then incubated under normal growth conditions for an additional 30 min and washed with 3 volumes of PBS, centrifuged as aforementioned, and decanted. This was repeated 2 additional times. Fixable Viability Dye eFluor^®^ 780 (eBiosciences, San Diego, CA, USA) 2 μL of 1:10 dilution in PBS) was added to the residual volume, briefly vortexed, and incubated in the dark at room temperature for 5 min. Cells were washed with 2 mL PBS, centrifuged, and decanted. Using the FoxP3/Transcription Factor Staining Buffer Set (eBiosciences, San Diego, CA, USA), cells were fixed and permeabilized by the addition of 1 mL of Fixation/Permeabilization Working Solution (eBiosciences, San Diego, CA, USA), briefly vortexed then incubated for 60 min at 4 °C in the dark. Samples were then washed 2 times with 2 mL of permeabilization buffer followed by 800*× g* for 5 min at 4 °C.

Antibodies containing the manufacturers’ recommended concentrations were added to the residual volume. Samples were incubated overnight (16 h) at 4 °C in the dark, then washed 2 times with 2 mL of permeabilization buffer followed by 800 *× g* for 5 min at 4 °C. Pellets were resuspended in 0.5 mL of PBS for FACS analysis. For the QuantaGene 2.0 Multiplex Assay, 2 *×* 10^6^ PBMCs were aliquoted to separate flow tubes after barcoding. Surface and viability staining was performed using the recommended test volumes for Fixable Viability Dye eFluor^®^ 780 (eBiosciences, San Diego, CA, USA), anti-CD3-PerCP (BioLegend, San Diego, CA, USA; clone OKT3), anti-CD4-PE (BioLegend, San Diego, CA, USA; clone OKT4), and anti-CD8-AF700 (BioLegend, San Diego, CA, USA; clone RPA-T8) on ice in the dark for 30 min (Table 1). Cells were washed with 2 mL of PBS, resuspended in Optimizer media at 5 *×* 10^6^ cells/mL and sorted into Optimizer media according to EtBr treatment. All gating strategies can be found in the Supplemental Material (Figures S1–S5).

### 2.5. JC-1 Staining

Immediately after the short-term pyocyanin treatment or twenty-four hours after the last long-term pyocyanin treatment, MUTZ-3 cells were collected and stained with JC-1 Mitochondrial Membrane Potential Assay Kit (Cayman Chemicals, Ann Arbor, MI, USA) according to the manufacturer’s protocol. Cells from the control groups were set aside for positive and negative CellROX^®^ controls as described above. JC-1 cells were sorted base on live cells, and processed according to the protocol used for MSCs, with the one substitution of CD34-PE to phenotype HPCs. The following day, at the completion of the RedOx staining protocol, cells were analyzed based on CD34 expression.

### 2.6. Quantitative PCR for Mitochondrial DNA Enumeration

PBMCs were sorted according to EtBr treatment for mtDNA quantification as described by Phillips et al. with minor modifications to accommodate the CFX96^™^ Real-Time PCR Detection System (Bio-Rad, Hercules, CA, USA) [20]. Two sets of mtDNA primers and probes were quantified and normalized to the single copy nuclear gene, RNaseP. Standards were constructed using gBlocks Gene Fragments (IDTDNA, Caralville, IA, USA) for each primer-probe pair (Table 2). For rhesus macaque MSC mtDNA quantification, separate primers and standards were prepared (Table 3). For quantifying PBMC mtDNA, reactions were prepared using 2X TaqMan^®^ Universal PCR Master Mix (Applied Biosystems, Foster City, CA, USA) Bio-, 50 nM mtMin primers, 50 nM mtMaj primers, 150 nM RNaseP primers, 200 nM mtMin probe, 200 nM mtMaj probe, 200 nM RNaseP probe, and 5 ng of sample. For MSC mtDNA quantification, reactions were prepared as listed for PBMCs with the exception of using 500 nM primer concentrations, and iTaq^™^ Universal SYBR^®^ Green Supermix (Bio-Rad, Hercules, CA, USA) was used in place of 2X TaqMan^®^ Universal PCR Master Mix. All oligonucleotides where purchased from IDTDNA (Coralville, IA, USA), except for the mtMin probe and mtMaj probe (Applied Biosystems, Foster City, CA, USA). Standard curves for PBMC mtDNA quantification were prepared by performing 2-fold serial dilutions from the highest standard (1.4 *×* 10^4^ copies/reaction for RNaseP; 2.7 *×* 10^6^ copies/reaction for both mtMin and mtMaj). Standard curves for MSC mtDNA quantification were prepared by performing 10-fold serial dilutions from the highest standard (2.63 *×* 10^7^ copies/reaction for RPLP0; 5.26 *×* 10^7^ copies/reaction for mtDNA). Thermocycling conditions were 95 °C for 10 min, followed by 40 cycles of 95 °C for 15 s, 55 °C for 15 s, and 60 °C for 1 min.

### 2.7. QuantiGene Plex Assay for mRNA Quantification

Live CD3+, CD8+ T cells were sorted based upon the barcoded, EtBr treatment group and positive control (3.5 *×* 10^5^ each treatment). A 19-plex QuantaGene 2.0 Multiplex Assay (ThermoFisher Scientific, Waltham, MA, USA) was developed to semi-quantify mRNA expression for mitohormetic and telomere related genes, along with 4 housekeeping genes (Table 4). Samples were processed according to the QuantiGene protocol supplied by the manufacturer. Data were acquired on a Luminex^®^ 200^™^, and gene expression was analyzed for samples in which at least 50 beads were counted for each sample. *RPLP0* was used to normalize target gene expression.

### 2.8. Animals, Gastric Catheter Implantation, and Alcohol Administration Protocol

This study was conducted at the Tulane National Primate Research Center (TNPRC) in Covington, Louisiana, on male rhesus monkeys (Macaca mulatta) of Indian origin that were 4 to 6 years of age. Institutional Animal Care and Use Committees at TNPRC and LSU Health Sciences Center (LSUHSC) in New Orleans approved experimental procedures on these animals. Data from a total of 24 animals were included in these analyses. Animals were fed a commercial primate chow, supplemented with fruit, and received alcohol (30% alcohol as a 0.5 h infusion) or isocaloric sucrose daily via a permanently indwelling intragastric catheter (17 gauge; Access Technologies, Skokie, IL, USA) that was attached to a cage-mounted swivel via a tether (Lomir Biomedical, Malone, NY, USA) as previously described [21]. A blood sample was obtained weekly 2 h after starting alcohol delivery in order to adjust infusion rates so that plasma alcohol concentrations were between 50 and 60 mM. Following 3 months of alcohol or sucrose administration, bone marrow aspirates were collected from the humeri for MSC isolation.

### 2.9. Bone Marrow Mononuclear Cell Isolation and MSC Culture and Enrichment

Bone marrow samples were collected from anesthetized animals following an overnight fast in the absence of detectable blood alcohol levels, and mononuclear cells were isolated to obtain mesenchymal stromal cells as previously described [22]. Briefly, mononuclear cells were collected using the Ficoll–Paque method and resuspended in α-MEM supplemented with 16.5% heat inactivated fetal bovine serum (h.i.FBS), 2% pen/strep, and 2 mM L-glutamine (L-glu; Complete Culture Medium (CCM). Cells (2 *×* 10^6^ cells/mL in 2 mL CCM) were plated onto prefilled (18 mL CCM) 15 cm tissue culture dishes followed by overnight culture in a humidified incubator (5% CO_2_) at 37 °C. After 24 h, plates were washed to remove nonadherent cells, followed by addition of 20 mL CCM. Cells are incubated until 60–70% confluent (5–10 d) with media changes every 2–3 d. Cells were trypsinized, washed, and cryopreserved at 2 *×* 10^6^ cells/mL for future expansion and studies.

For RedOx flow experiments, passage 2 MSCs isolated from control and CBA-administered macaques were seeded in 10 cm cell culture dishes at 5 *×* 10^5^ cells per 10 cm cell culture plate. At this point, the two groups of MSCs were divided into in vitro 0 and 50 mM alcohol groups and cultured for 3 d in a humidified incubator containing alcohol in the water bath that maintains the desired media alcohol concentration as previously described [23].

### 2.10. Statistical Analysis

Data are presented as mean ± standard error of the mean (SEM). The sample size is indicated in the legend of each figure. Statistical analyses of data were performed using GraphPad Prism 5 (GraphPad Software, Inc., La Jolla, CA, USA). Unpaired Student’s *t*-test (for comparison between 2 groups) or two-way ANOVA followed by Tukey’s posttest (for comparisons among multiple groups) were conducted. Pearson’s correlation analysis was performed for mean fluorescence intensity ratios. Differences were considered statistically significant at *p* < 0.05.

## 3. Results

### 3.1. Short-Term In Vitro Oxidative Stress in MUTZ-3 Hematopoietic Progenitor Cells

**Figure 2.**
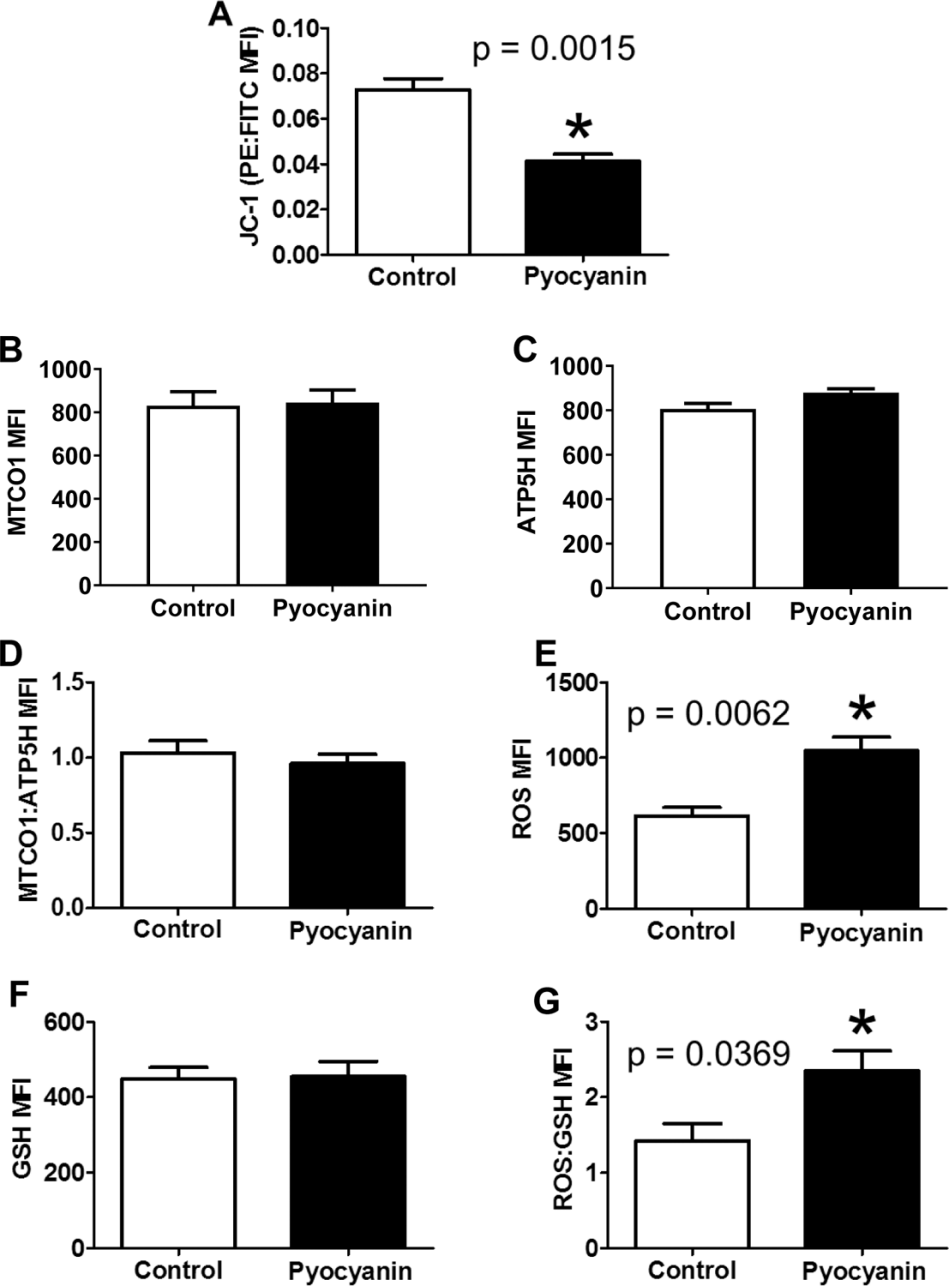
Acute (16hr) pyocyanin treatment effects on mitochondrial polarization, mitochondrial genomic stability, and redox parameters. (A) Control cells were significantly more polarized than pyocyanin-treated (10μM) cells. (B) Mitochondrial genome stability was not different between groups. (C) Total cellular ROS was significantly increased by pyocyanin. (D) Reduced glutathione was not significantly different between groups. (E) Oxidative burden was significantly increased by pyocyanin treatment. Data are expressed as mean ± SEM. * = p < 0.05 as determined by Student’s unpaired t-test; n=4

After treatment with 10 μM pyocyanin for 16 h, MUTZ-3 cell JC-1 staining revealed significantly depolarized mitochondria (Figure 2A). Mitochondrial genomic integrity was assessed by calculating the ratio of median fluorescence intensities for MTCO1 and ATP5H, did not show a difference between control and treated samples (Figure 2B–D).

**Figure 3.**
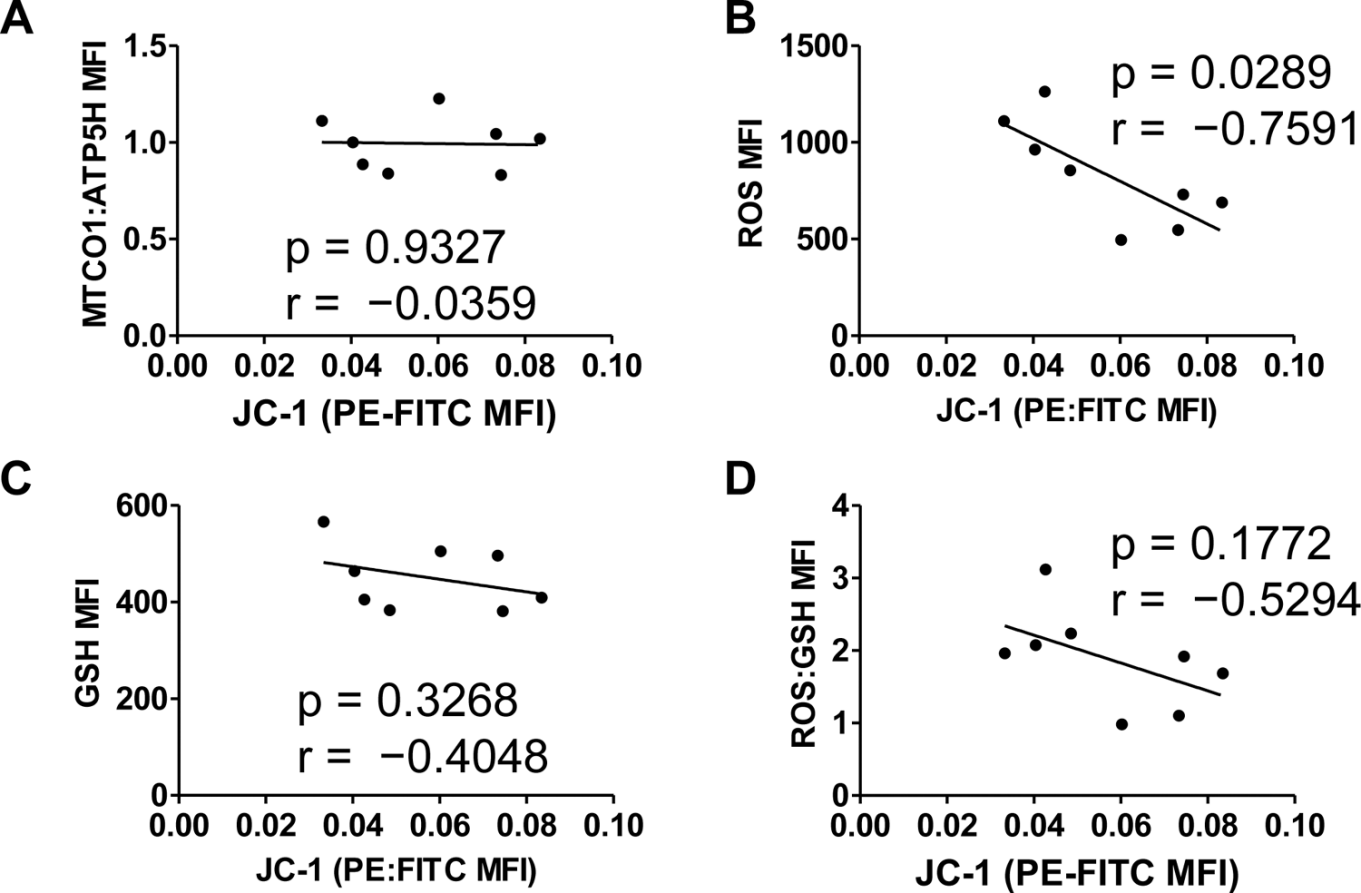
Correlations of mitochondrial genomic stability and redox parameters to mitochondrial polarization state. (A) Mitochondrial polarization was not correlated to mitochondrial genomic stability. (B) Total cellular ROS negatively correlated to mitochondrial depolarization. Neither reduced glutathione (C) nor oxidative burden (D) correlated to mitochondrial polarization status. Pearson’s test for correlation; N = 8.

Total cellular ROS was increased after pyocyanin treatment, while reduced glutathione was unchanged (Figure 2E,F). Calculated oxidative burden, the ratio of median fluorescence intensities for CellRox and GSH, showed an increase in total oxidative burden (Figure 2G). Mitochondrial polarization was not correlated to mitochondrial genomic stability (Figure 3A). As expected, total cellular ROS negatively correlated to mitochondrial depolarization (Figure 3B); however, reduced glutathione and oxidative burden did not correlate with mitochondrial polarization status (Figure 3C,D).

### 3.2. Long-Term In Vitro Oxidative Stress in MUTZ-3 Hematopoietic Progenitor Cells

**Figure 4.**
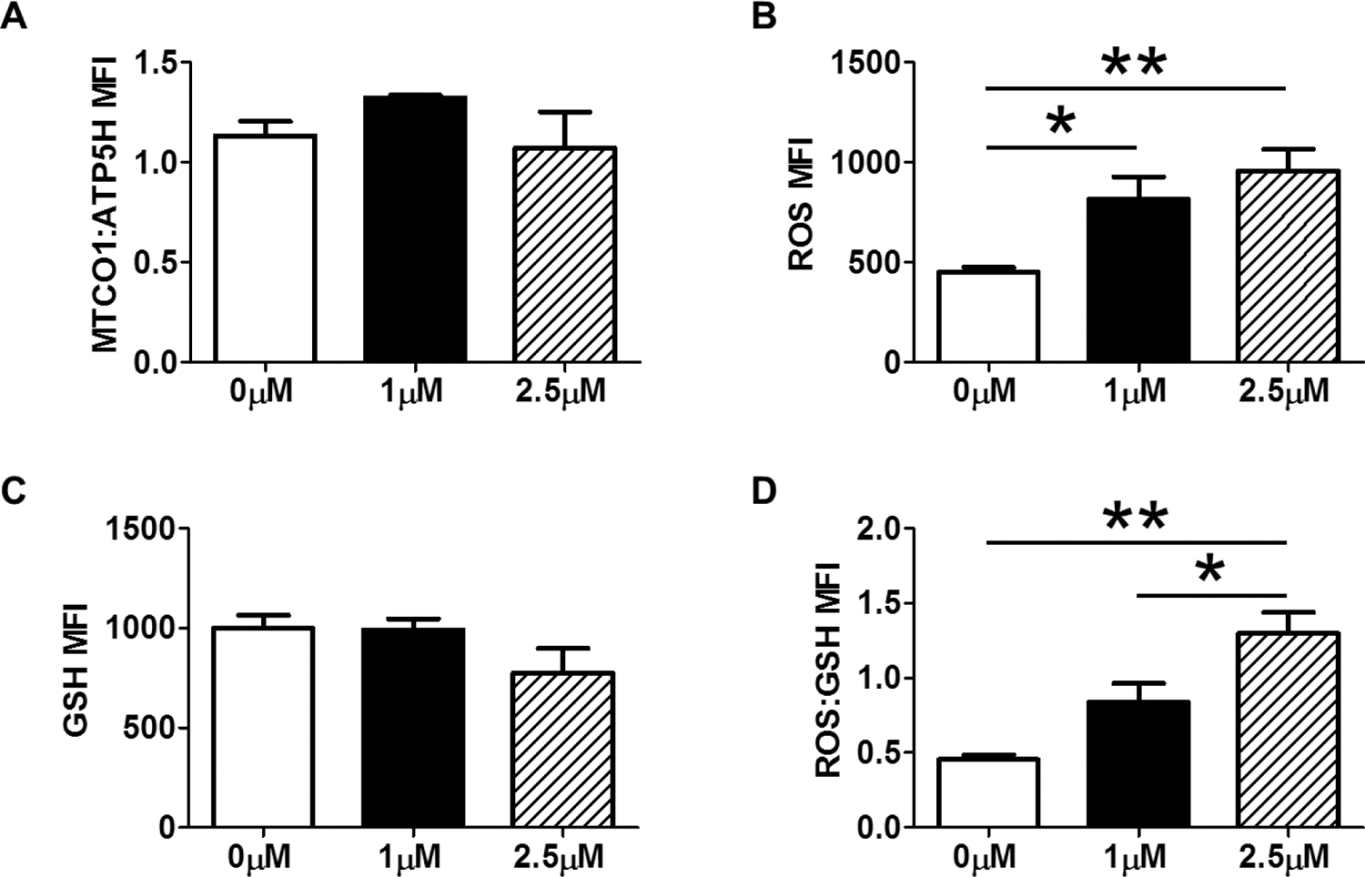
Chronic (9d) pyocyanin (1 & 2.5μM) treatment effects on mitochondrial polarization, mitochondrial genomic stability, and redox parameters (A) There were no differences in mitochondrial genomic stability among any treatment groups. (B) Total cellular ROS was significantly increased in both 1 and 2.5μM pyocyanin treatment groups versus control. (C) Reduced glutathione was not different among treatment groups. (D). Chronic pyocanin treatment dose-dependently increased total oxidative burden. Data are expressed as mean ± SEM. * = p < 0.05, ** = p < 0.01 as determined by One-way ANOVA followed by Tukey’s Multiple Comparison Test; n = 4 per group.

MUTZ3 CD34+ cells treated with pyocyanin for 12 days at concentrations of 5 μM or greater lead to significant cell death. Therefore, we chose 1 and 2.5 μM concentrations as low and high oxidative stress, respectively. We did not observe a change in mitochondrial genomic stability from control with either concentration after long-term treatment (Figure 4A). Our results showed an increase in ROS at both doses, with no statistically different changes in GSH (Figure 4B,C). We did observe a dose-dependent increase in total oxidative burden due to long-term pyocyanin treatment (Figure 4D).

### 3.3. T Cell Antioxidant Status, Mitochondrial Genomic Stability, mtDNA Quantification and Gene Expression Analysis

**Figure 5.**
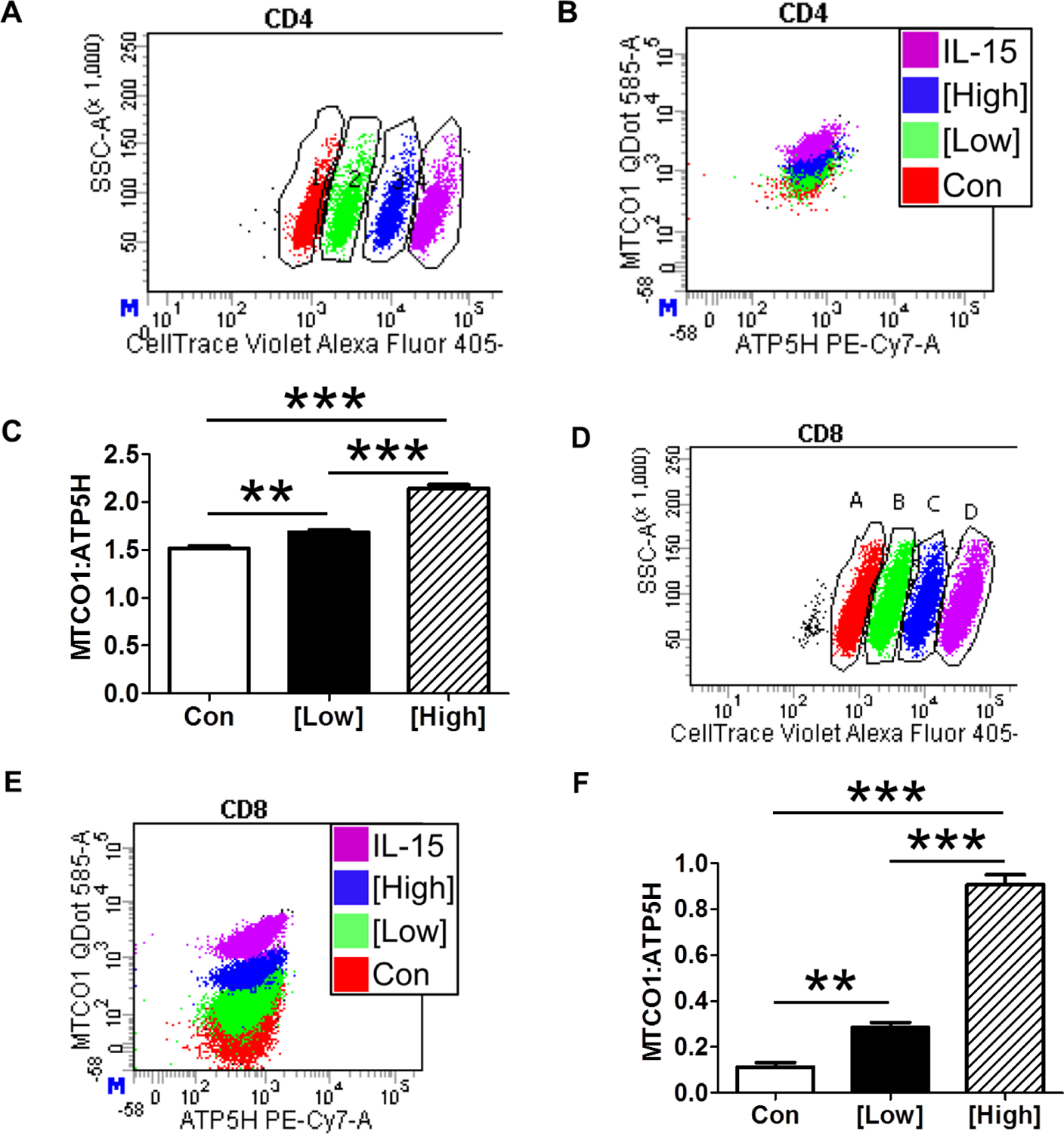
Chronic (3 days) ethidium bromide (EtBr) treatment of PBMCs. (A) Representative flow plot of barcoded CD4+ T lymphocytes after gating on live, CD3+CD4+ cells. (B) Representative flow plot of mitochondrial genomic stability parameters gated from A. (C) EtBr dose-dependently increases CD4 T Cell mitochondrial genomic stability. (D) Representative flow plot of barcoded CD8+ T lymphocytes after gating on live, CD3+CD8+ cells. (E) Representative flow plot of mitochondrial genomic stability parameters gated from D. (F) EtBr dose-dependently increases CD8 T Cell mitochondrial genomic stability. There were no effects of EtBr treatment on ROS, glutathione or oxidative burden. Data are expressed as mean ± SEM. ** = p < 0.01, *** = p < 0.001 by One-way ANOVA followed by Tukey’s Multiple Comparison Test; [Low] = 400 ng/mL; [High] = 1,200 ng/mL; n = 4 per group.

**Figure 6.**
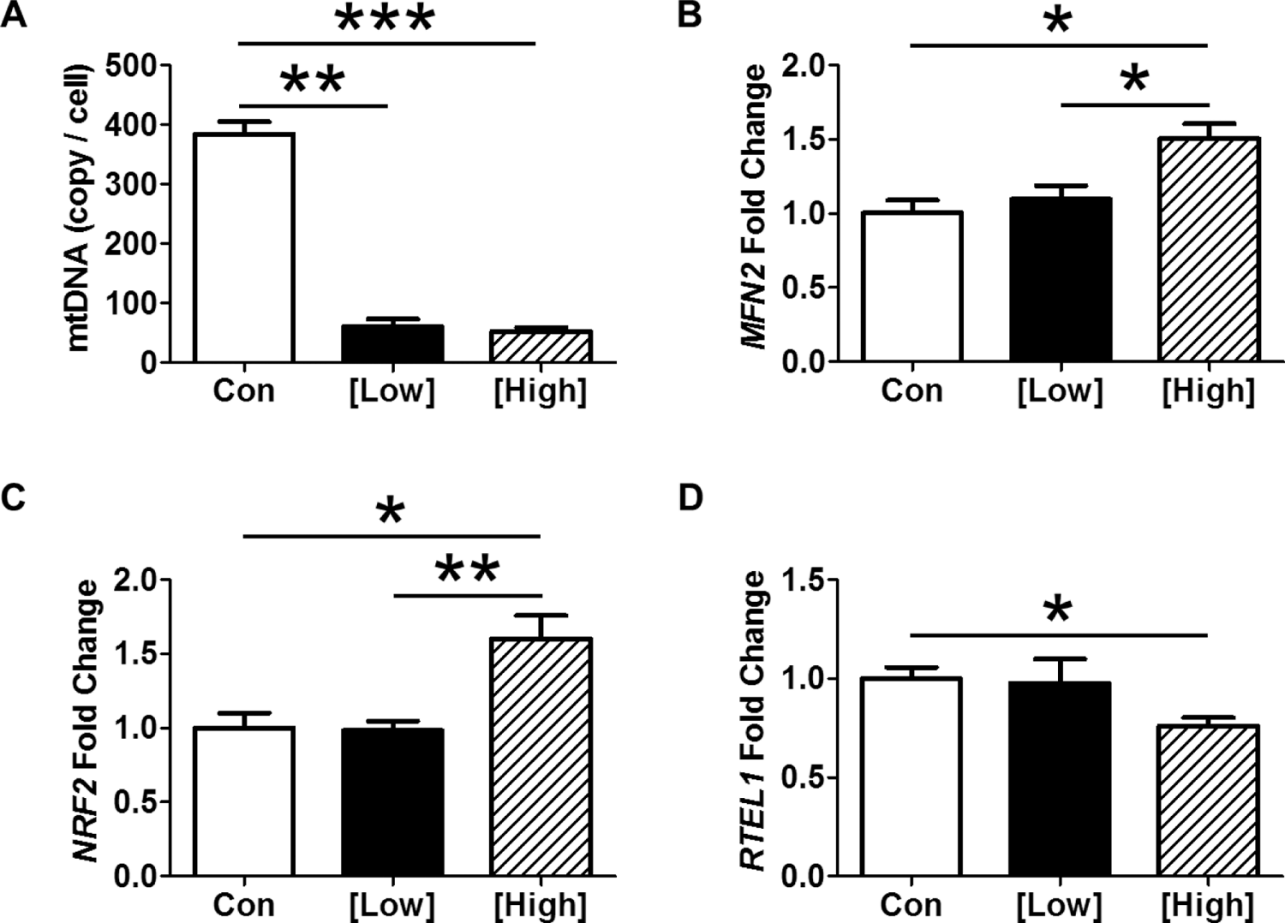
CD8+ T lymphocytes were sorted according to treatment groups. (A) Both low and high dose EtBr treatment depleted mtDNA to the same extent. High dose EtBr increased mRNA gene expression of (B) *MFN2*, (C) *NRF2*, and (D) *RTEL1*. Data are expressed as mean ± SEM. * = p < 0.05, ** = p < 0.01, *** = p < 0.001 by One-way ANOVA followed by Tukey’s Multiple Comparison Test; [Low] = 400 ng/mL; [High] = 1,200 ng/mL; n = 4 per group.

PBMCs were treated with anti-CD3/anti-CD28 dynabeads to stimulate T cell proliferation and treated with 400 and 1200 ng/mL ethidium bromide to deplete mitochondrial DNA [24]. PBMCs were barcoded by treatment groups (Figure 5A,D). Surprisingly live CD3+, CD4+ T cells had increased MTCO1:ATP5H ratios as seen in Figure 5B. EtBr dose-dependently increased CD4+ T lymphocyte mitochondrial genomic integrity (Figure 5C). There were no effects of EtBr treatment on ROS, GSH, or oxidative burden in CD4+ T cells. Analysis of CD3+, CD8+ T cells also showed an increased MTCO1:ATP5H ratio, and increased mitochondrial genomic integrity (Figure 5E,F). Similar to CD4+ T cells, there were no effects of EtBr treatment on ROS, GSH, or oxidative burden in CD8+ T cells. After sorting CD8+ T cells based upon treatment group, we unexpectedly found that both low- and high-dose EtBr treatment depleted mtDNA (copy’s/cell) to the same extent (Figure 6A).

High-dose EtBr increased mRNA gene expression of *MFN2* over both control and low-dose EtBr (Figure 6B), indicative of activation of the mitochondrial fusion machinery. Mitochondrial fusion is the primary mechanism by which cells preserve mitochondrial function, rather than a true DNA repair mechanism [25]. *NRF2* is a ReDox sensitive gene that stimulates glutathione synthase expression [26]. High-dose EtBr increased NRF2 over both control and low-dose groups (Figure 6C). ROS have also been suggested to play a significant role in biological aging. Since CD8+ T lymphocytes show decreased telomere length with biological aging and chronic stressors, we examined *RTEL1* expression [27–30]. Only high-dose EtBr treatment decreased *RTEL1* mRNA expression (Figure 6D).

### 3.4. Effect of CBA and In Vitro Alcohol on MSCs and Oxidative Burden

**Figure 7.**
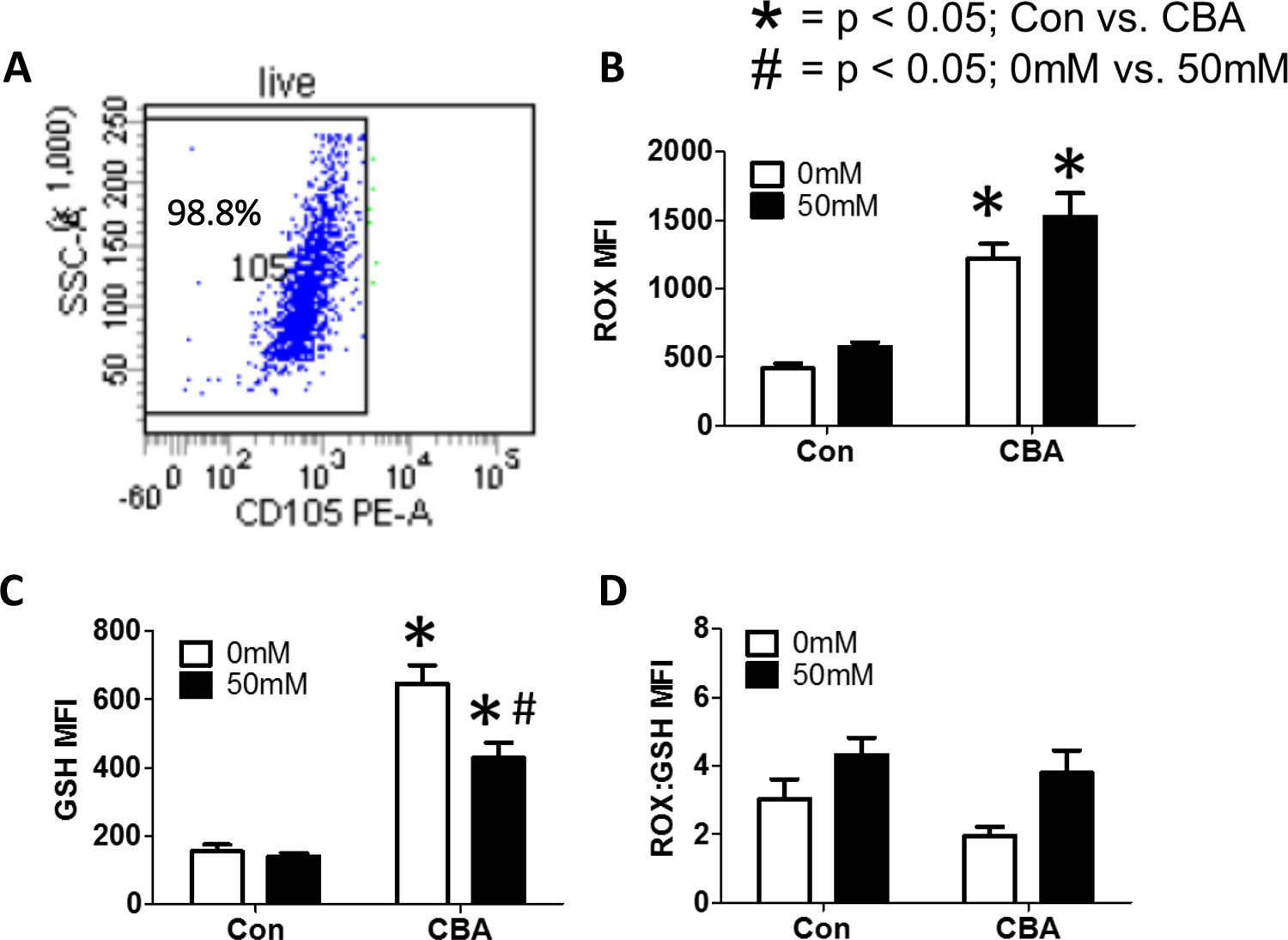
Chronic (3 days) *in vitro* ethanol treatment of MSCs isolated from control and CBA-administered rhesus macaques. (A) Representative flow plot of CD105+ MSCs after gating on live cells. (B) *In vivo* CBA increases ROS compared to control, regardless of ethanol treatment. (C) *In vivo* CBA increases GSH compared to control. *In vitro* ethanol (50mM) treatment decreased GSH in MSCs isolated from CBA-administered macaques. (D) *In vivo* CBA administration and *in vitro* 50mM ethanol had no significant effect on ROS:GSH in MSCs isolated from sucrose and CBA-administered rhesus macaques. Data are expressed as mean ± SEM. * = p < 0.05 for Con vs. CBA, # = p < 0.05 for 0mM vs. 50mM by Two-way ANOVA followed by Bonferroni’s posthoc test; n = 5 per group. Abbreviations: Con = Control, CBA = Chronic Binge Alcohol.

MSCs isolated from control and chronic binge alcohol (CBA)-administered macaques (Figure 7A) were treated in vitro with or without 50 mM alcohol for 72 h. Following this treatment, cells were harvested to assess in vivo and in vitro effects of alcohol on RedOx parameters, as well as mitochondrial genomic stability. In vivo CBA increases ROS in MSCs isolated from CBA-administered rhesus macaques compared to control, regardless of in vitro ethanol treatment (Figure 7B). In vivo CBA increases GSH in MSCs isolated from CBA-administered rhesus macaques compared to control and in vitro ethanol (50 mM) treatment decreased GSH in MSCs isolated from CBA-administered macaques (Figure 7C). In vivo CBA administration and in vitro 50 mM ethanol had no significant effect on ROX:GSH in MSCs isolated from sucrose and CBA-administered rhesus macaques (Figure 7D).

**Figure 8.**
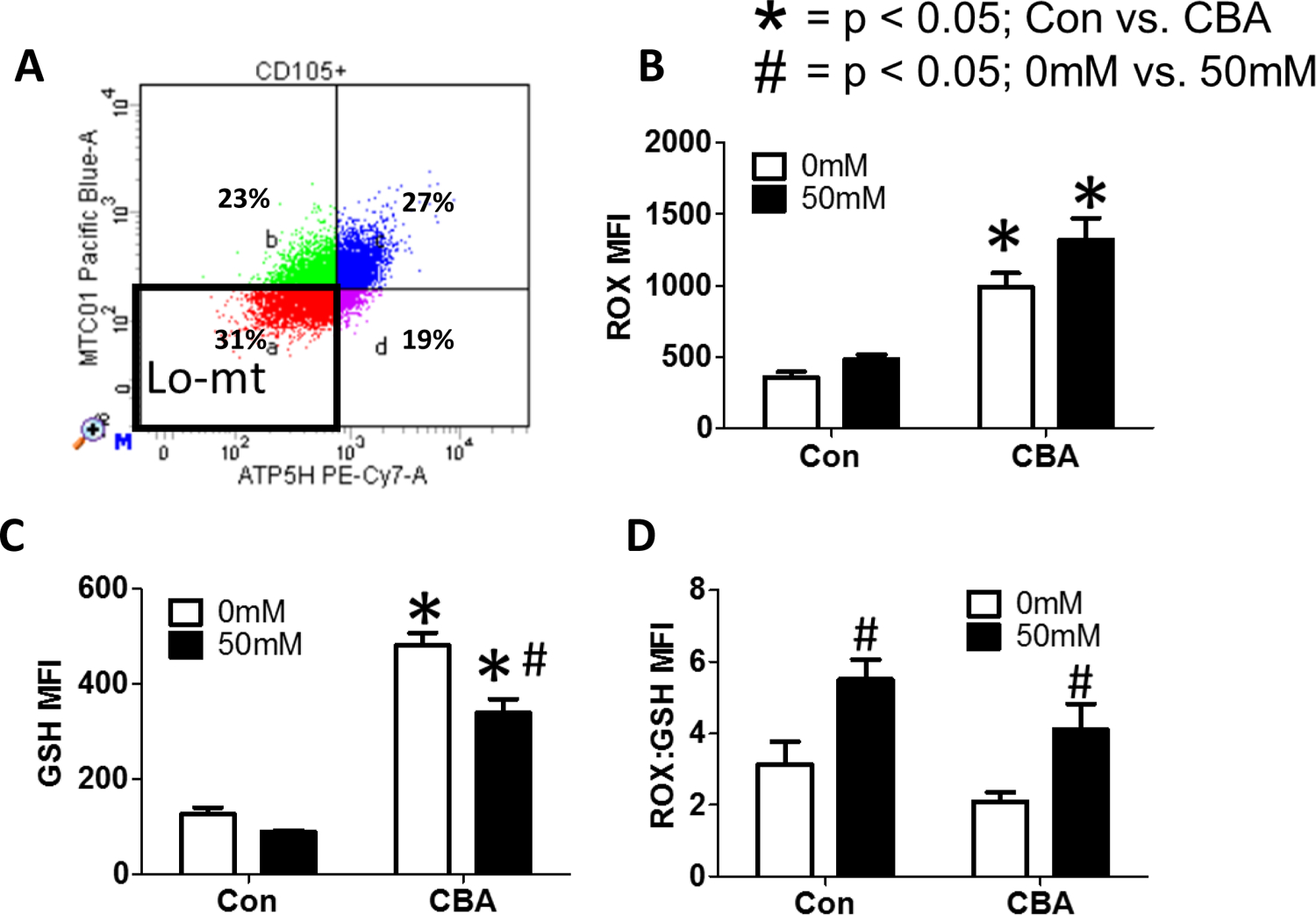
Chronic (3d) *in vitro* ethanol treatment of MSCs with low mitochondrial content isolated from control and CBA-administered rhesus macaques. (A) Representative flow plot of MSCs with low mitochondrial content after gating on live, CD105+ cells. (B) *In vivo* CBA increases ROS, regardless of *in vitro* ethanol treatment. (C) *In vivo* CBA increases GSH compared to control. *In vitro* ethanol (50mM) treatment decreased GSH in MSCs isolated from CBA-administered macaques. (D) *In vivo* CBA administration had no significant effect on ROS:GSH, while *in vitro* 50mM ethanol increased ROS:GSH in MSCs isolated from sucrose and CBA-administered rhesus macaques. Data are expressed as mean ± SEM. * = p < 0.05 for Con vs. CBA, # = p < 0.05 for 0mM vs. 50mM by Two-way ANOVA followed by Bonferroni’s posthoc tests; n = 5 per group. Abbreviations: Con = Control, CBA = Chronic Binge Alcohol.

After sorting MSC cells for low mitochondrial content (Figure 8A), we found that in vivo CBA increases ROS in MSCs isolated from CBA-administered rhesus macaques compared to control, regardless of in vitro ethanol treatment (Figure 8B). In vivo CBA increases GSH in MSCs isolated from CBA-administered rhesus macaques compared to control and in vitro ethanol (50 mM) treatment decreased GSH in MSCs isolated from CBA-administered macaques (Figure 8C). In vivo CBA administration had no significant effect on ROX:GSH, while in vitro 50 mM ethanol increased ROX:GSH in MSCs isolated from sucrose and CBA-administered rhesus macaques (Figure 8D).

**Figure 9.**
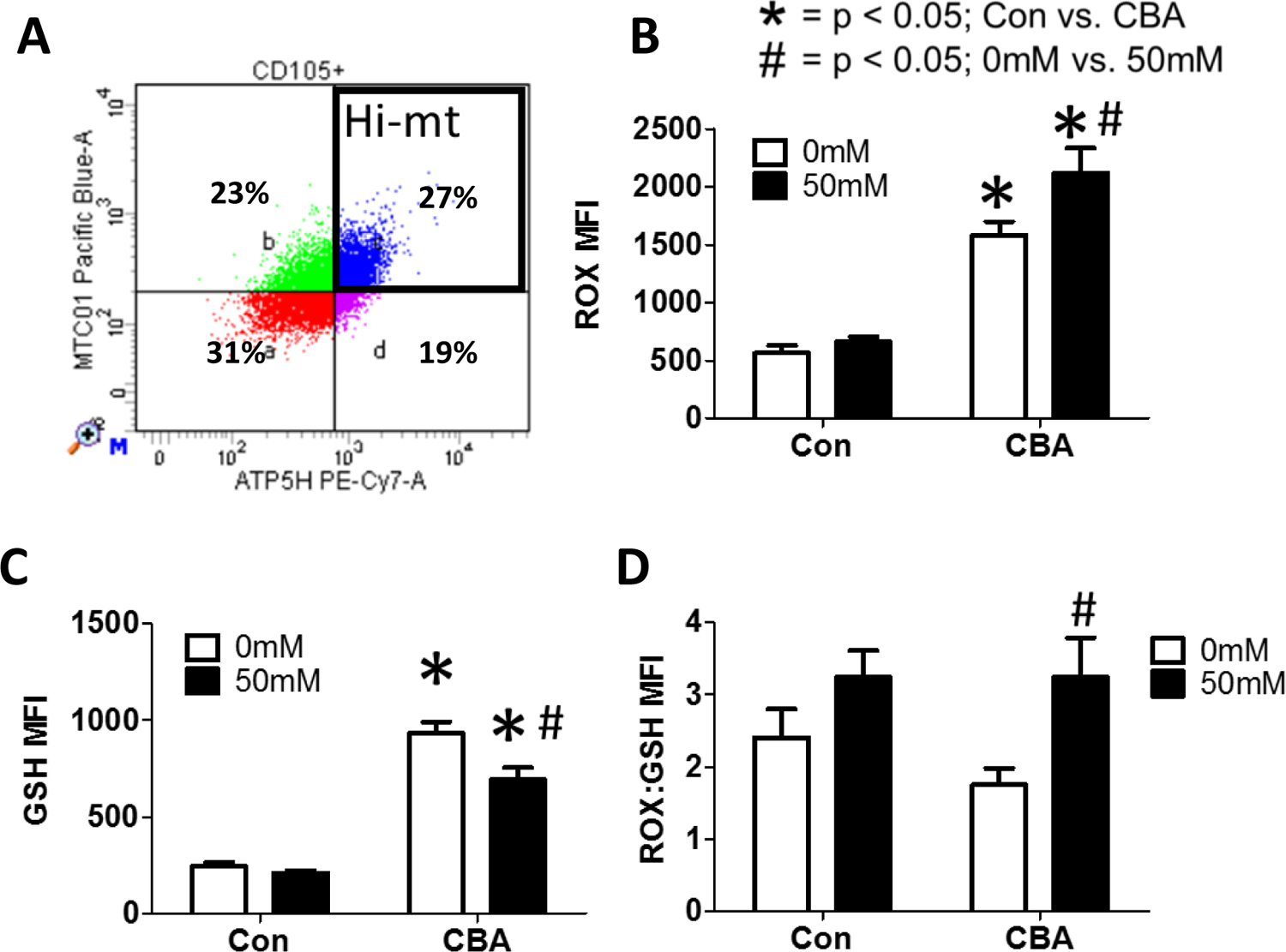
Chronic (3 days) *in vitro* ethanol treatment of MSCs with high mitochondrial content isolated from control and CBA-administered rhesus macaques. (A) Representative flow plot of MSCs with high mitochondrial content after gating on live, CD105+ cells. (B) *In vivo* CBA increases ROS compared to control. *In vitro* ethanol (50mM) treatment further increased ROS in MSCs isolated from CBA-administered macaques. (C) *In vivo* CBA increases GSH compared to control. *In vitro* ethanol (50mM) treatment decreased GSH in MSCs isolated from CBA-administered macaques. (D) *In vivo* CBA administration had no significant effect while *in vitro* 50mM ethanol increased ROS:GSH in MSCs isolated from CBA-administered rhesus macaques. Data are expressed as mean ± SEM. * = p < 0.05 for Con vs. CBA, # = p < 0.05 for 0mM vs. 50mM by Two-way ANOVA followed by Bonferroni’s posthoc tests; n = 5 per group. Abbreviations: Con = Control, CBA = Chronic Binge Alcohol

We also analyzed MSCs with high mitochondrial content for these same parameters (Figure 9A). In vivo CBA increases ROS in MSCs isolated from CBA-administered rhesus macaques compared to control and in vitro ethanol (50 mM) treatment further increased ROS in MSCs isolated from CBA-administered macaques (Figure 9B). In vivo CBA increases GSH in MSCs isolated from CBA-administered rhesus macaques compared to control and in vitro ethanol (50 mM) treatment decreased GSH in MSCs isolated from CBA-administered macaques (Figure 9C). In vivo CBA administration had no significant effect while in vitro 50 mM ethanol increased ROX:GSH in MSCs isolated from CBA-administered rhesus macaques (Figure 9D).

### 3.5. Mitochondrial DNA Quantification and Indication of Mitochondrial Genomic Stability

**Figure 10.**
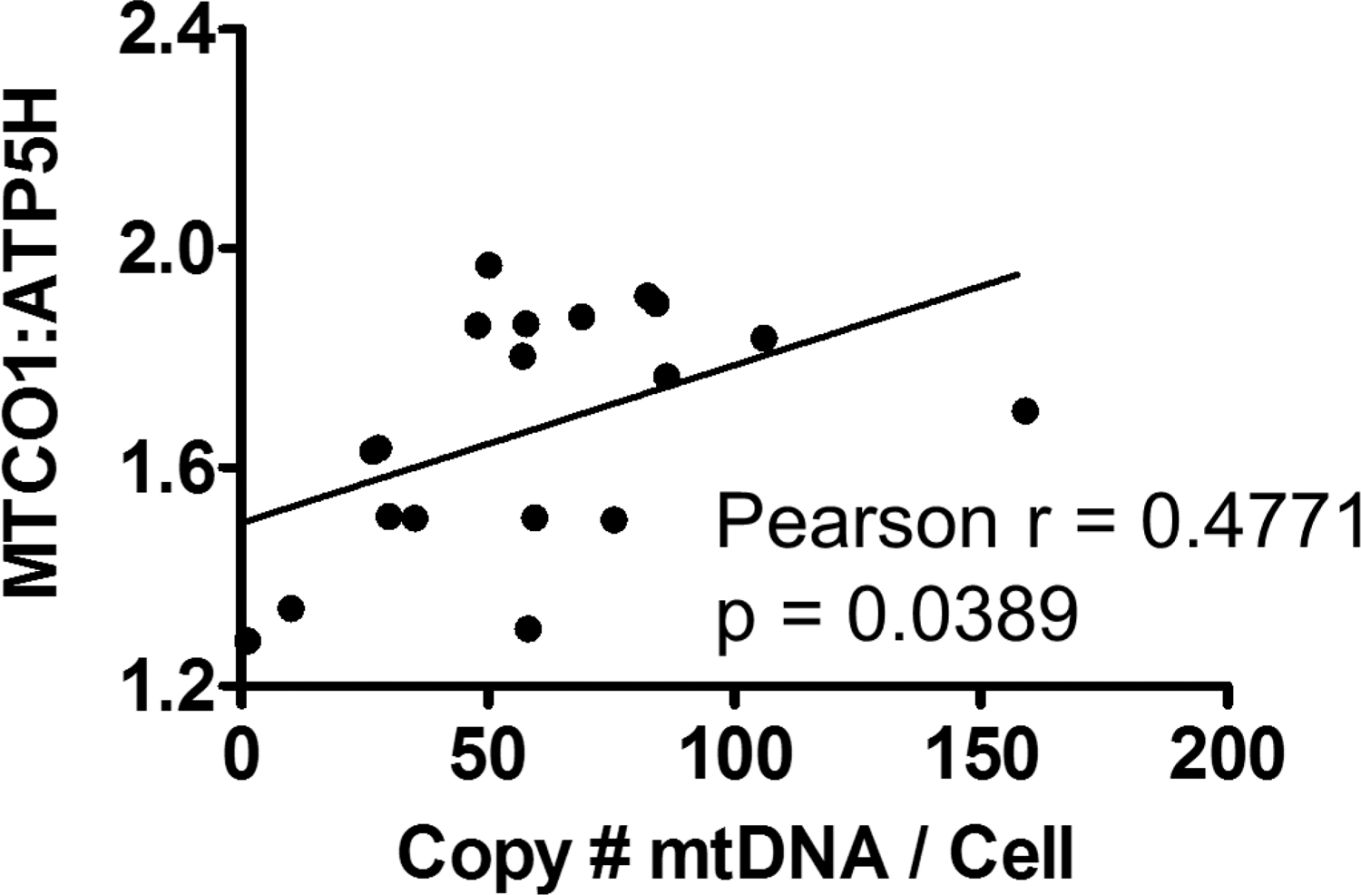
Mitochondrial DNA content positively correlates to mitochondrial genomic stability in MSCs isolated from Control and CBA-administered rhesus macaques treated with 0 or 50mM *in vitro* ethanol (3 days). r = Pearson’s correlation; n = 20.

MSC Cells were sorted based on low versus high mitochondrial content and subjected to qPCR for mtDNA. MtDNA quantity was not different among any of the groups.This assay measures total mtDNA, and does not discriminate between health and damaged mtDNA. Additionally, MSCs are undifferentiated and contain few mtDNA genomes per cell. Nevertheless, there was a positive correlation between the ratio of MTCO1:ATP5H MFIs to mtDNA content (Figure 10). This was surprising, given that the sorted MSCs did not have different MTCO1:ATP5H MFI ratios, regardless of in vivo or in vitro ethanol treatment.

## 4. Discussion

The novel workflow presented in this manuscript simultaneously measures mtDNA integrity (indicator of genomic stability) and RedOx status (by measuring reduced glutathione and reactive oxygen species). This assessment is novel and highly beneficial compared to previous techniques [5–13], as it does not rely on parallel measurements and is streamlined to give a readout of ROS and mitochondrial genomic stability of a single cell. Further, this workflow can be used on sorted cell populations and can be combined for further downstream analysis. With up to 15 channels available, this allows for addition of other genetically encoded probes for ROS or proteins of interest.

RedOx signaling is widely accepted as being a major regulator of the hematopoietic niche [31,32]. The MUTZ-3 cell line model for hematopoietic stem and progenitor cells was utilized in this study. To assess the utility of our ReDox measurement parameters, we induced acute oxidative stress in MUTZ-3 cells with pyocyanin. Pyocyanin is a protein isolated from *Pseudomonas aeruginosa* that increases H_2_O_2_ production in cell cultures [16]. While pyocyanin did induce ROS accumulation in MUTZ-3 cells, short-term treatment did not alter mitochondrial genomic stability (MTCO1:ATP5H MFI ratio). We hypothesized that the treatment duration was too short to allow for MTCO1 protein degradation. Therefore, we pyocyanin treated MUTZ3 cells for a longer term with the expectation of decreasing mitochondrial genomic stability. While there was a pyocyanin dose-dependent increase in cellular ROS, we did not detect damage to the mitochondria as assayed by the MTCO1:ATP5H ratio. We believe this was due to either (1) the culture media for MUTZ-3 cells containing a significant amount of glutathione, (2) CD34+ cells and transformed cells being less reliant on mitochondria for energy production, or (3) a combination of these factors [33,34]. Further, we predict that longer treatments with pyocyanin would deplete glutathione and lead to mitochondrial damage in MUTZ-3 cells, as observed in our alcohol studies using MSCs.

We treated human PBMCs with ethidium bromide (EtBr), to deplete mitochondria. EtBr intercalates into mtDNA and prevents replication [24]. As cells divide, mtDNA is diluted from successive generations. In our culture system, EtBr treatments began when the PBMCs were maximally dividing. The doses and timing were empirically determined. We expected a dose-dependent decrease in mitochondrial genomic stability (MTCO1:ATP5H MFI ratio); however, we observed the opposite effect. While we did see an overall increase in mitochondrial genomic stability, mtDNA was severely depleted in cultures at either the low- or high-dose EtBr to the same extent. We hypothesize that EtBr treatment may have induced a protective, mitohormetic response. In support of this finding, there was an increase in *Mitofusin 2 (MFN2)* expression. This suggests that while the DNA was markedly depleted, the fusion machinery maintained stable mitochondria over the duration of these experiments [35,36]. An alternative explanation could be that mitophagy eliminated damaged mitochondria, preventing increases in oxidative stress [37,38]. Increased expression of *Nuclear respiratory factor 2 (NRF2)* in the high-dose EtBr-treated cells suggests that these cultures were beginning to sense increased ROS, as ROS stimulates *NRF2* expression [39]. Finally, ROS have been suggested to play a significant role in biological aging. A common marker of aging is decreased telomere length [40]. Telomere attrition is most pronounced in CD8 T lymphocytes [27–30]. We found that the high-dose EtBr groups decreased *Regulator of telomere elongation helicase 1 (RTEL1)* expression. We hypothesize that while we see what appears to be a mitohormetic response at both doses of EtBr, the high-dose treatment causes allostatic overload in these cultures [17].

Finally, we examined mesenchymal stromal cells (MSCs) isolated from rhesus macaques, isolated after 3 months of isocaloric sucrose (control) or chronic binge alcohol (CBA) administration. Chronic alcohol induces a pro-oxidative milieu in most tissues that have been examined [41]. Additionally, MSCs have been shown to maintain their in vivo properties for up to five passages ex vivo [42]. Using our workflow, we found that in vivo CBA administration increased total MSC ROS when compared to control MSCs. This was accompanied by a concomitant increase in GSH, suggesting a new allostatic set point in MSCs isolated from CBA macaques. Interestingly, when challenged with in vitro alcohol, MSCs from control monkeys did not show any appreciable change in either ROS or GSH content. The CBA MSCs had further increases in ROS with in vitro alcohol treatment, but a decline in GSH. This may suggest allostatic overload in this model system. Finally, analysis of the MTCO1:ATP5H MFI ratio was not statistically different among the groups, but the ratio did correlate to mtDNA quantity. It should be noted that we did not sort cells based on MTCO1:ATP5H ratio, so this correlation was surprising and would be expected to be greater had we sorted cells based on this mitochondrial genomic stability parameter. Additionally, MTCO1:ATP5H declines with age, and disease [43]. The macaques in this study were all adolescent, and had only been challenged with 3 months of CBA. Further studies are underway to examine the combined effects of simian immunodeficiency virus and antiretroviral therapy on MSCs RedOx status and mitochondrial genomic stability.

From our experiments using PBMCS and MSCs, we found that the MTCO1:ATP5H ratio correlated positively with mtDNA quantity in MSCs (Figure 10), but saw the opposite result in PBMCs. We believe these inconsistencies are due to differences in heterogeneity within these cell cultures. PBMCs consist of many different cell types in different differentiation stages that could influence their mitochondria. For example, memory T cells have been observed to harbor significantly higher mitochondrial spare respiratory capacity (SRC) compared to naïve T cells, and this SRC correlates with enhanced mitochondrial biogenesis and expression of mitochondrial genes involved with the electron transport chain (19). Additionally, mitophagy is critical for the survival of memory T cells and has been shown to serve a protective role in their differentiation [44]. We predict these factors may contribute to the increased MTCO1 expression in ethidium bromide-treated PBMCs. With this understanding, further studies will be done to optimize this assay for PBMCs, as well as other cell types, to further the application of our novel FACS-based workflow in mitochondrial-health related studies.

## 5. Conclusions

In conclusion, we have developed a unique, powerful FACS-based workflow allowing for phenotype-specific RedOx multi-parameter cell isolation and analysis. The isolated cells can then undergo mtDNA quantification or gene expression analyses on multiple RedOx-sensitive pathways, depending on the downstream users’ pathways of interest. This workflow has the potential to advance all research investigating cellular RedOx status and mitochondrial stability that relies on knowledge of specific cell populations.

## Supplementary Material

Supplemental Material (Zip File)

## Figures and Tables

**Table 1.** Flow Cytometry Panels

**Panel for MSCs**	
ATP5H	PE_Cy7-ATP5H
MTCO1	Qd655-MTCO1
GSH	APC-GSH
CD105	PE-CD105
**Panel for HSPCs**	
ATP5H	PE_Cy7-ATP5H
MTCO1	Qd655-MTCO1
GSH	APC-GSH
CD34	PE-CD34
**Panel for T Cells**	
ATP5H	PE_Cy7-ATP5H
MTCO1	Qd655-MTCO1
GSH	APC-GSH
CD3	PerCP-CD3
CD4	PE-CD4
CD8	AF700-CD8

**Table 2.** Primers, probes, and gBlock standards used to quantify human mtDNA.

**Primer Pair & Probe 1**	
mtMin Forward	CTAAATAGCCCACACGTTCCC
mtMin Reverse	AGAGCTCCCGTGAGTGGTTA
mtMin Probe	6FAM/CATCACGATGGATCACAGGT/MGBNFQ
**Primer Pair & Probe 2**	
mtMaj Forward	CTGTTCCCCAACCTTTTCCT
mtMaj Reverse	CCATGATTGTGAGGGGTAGG
mtMaj Probe	VIC/GACCCCCTAACAACCCCC/MGBNFQ
**Housekeeping Gene**	
RNaseP Forward	AGATTTGGACCTGCGAGCG
RNaseP Reverse	GAGCGGCTGTCTCCACAAGT
RNaseP Probe	5Cy5/TTCTGACCTTAOGAAGGCTCTGCGCG/3IAbRQSp
**Gene Block Standards**	
mtMin	TCCTACTTCAGGGTCATAAAGCCTAAATAGCCCACACGTTCCCCTTAAATAAGACATCACGATGGATCACAGGTCTATCACCCTATTAACCACTCACGGGAGCTCTCCATGCATTTGGTATTTTCGTCT
mtMaj	AATCAACAACAACCTATTTAGCTGTTCCCCAACCTTTTCCTCCGACCCCCTAACAACCCCCCTCCTAATACTAACTACCTGACTCCTACCCCTCACAATCATGGCAAGCCAACGCCACTTATCCAGTGA
RNaseP	ATGGCGGTGTTTGCAGATTTGGACCTGCGAGCGGGTTCTGACCTGAAGGCTCTGCGCGGACTTGTGGAGACAGCCGCTCACCGTGAGTTGCCCCGGCTTCGCGCCTGGCCAACCTCATGCCACCC

**Table 3.** Primers and standards used to quantify rhesus mtDNA

**Primer Pair & Probe 1**	
mtDNA Forward	GTGAACTTGCCCTCGTAGTATAA
mtDNA Reverse	GGTGGTGGAGTTAGGTACTTT
**Housekeeping Gene**	
RPLP0 Forward	CAGCAAGTGGGAAGGTGTAATCC
RPLP0 Reverse	CCCATTCTATCATCAACGGGTACAA
**Standards**	
mtDNA	GTGAACTTGCCCTCGTAGTATAAATTAGTACACTGGCCTTGTAAACCAGAAATGAACACTCTTCCTAGGGCAGTCAGAAAGAAAGTACCTAACTCCACCACC
RPLP0	ACCCCATTCTATCATCAACGGGTACAAACGAGTCCTGGCCTTGTCTGTGGAGACGGATTACACCTTCCCACTTGCTGAA

**Table 4.** List of genes examined using QuantiGene 2.0 assay.

Gene Symbol	Name
**Mitochondrial DNA & Protein Maintenance**	
*MFN1* & *MFN2*	Mitofusin 1 & 2
*TFAM*	Transcription Factor A, mitochondrial
**Regulators of Oxidative Stress**	
*NOS1, NOS2*, & *NOS3*	Nitric Oxide Synthase 1, 2, & 3
*NRF1* & *NRF2*	Nuclear Respiratory Factor 1 & 2
*PPARGC1A* & *PPARGC1B*	PPAR-γ Co-activator α & β
*UCP1, UCP2*, & *UCP3*	Uncoupling Protein 1, 2, & 3
**Telomere Maintenance**	
*RTEL1*	Regulator of Telomere Elongation Helicase
*TERT*	Telomerase Reverse Transcriptase
**House Keeping Genes**	
*B2M*	Beta-2-microglobulin
*HPRT1*	Hypoxanthine Phosphoribosyltransferase 1
*RPLP0*	Ribosomal Protein, Large, P0
*TBP*	TATA-box Binding Protein

## Data Availability

The data presented in this study are available on request from the corresponding author.
