# Supplementary material for: A Novel FACS-Based Workflow for Simultaneous Assessment of RedOx Status, Cellular Phenotype, and Mitochondrial Genome Stability
Source: Biochem (Basel). Author manuscript; Available in PMC 2022 Aug 5. (PMC9355044; doi:10.3390/biochem1010001)
Supplement: Supplemental Material (Zip File) [file NIHMS1822005-supplement-Supplemental_Material__Zip_File_.zip › Supplemental_Figures_Biochem/Supplemental Figure 1.pptx]

## Slide 1
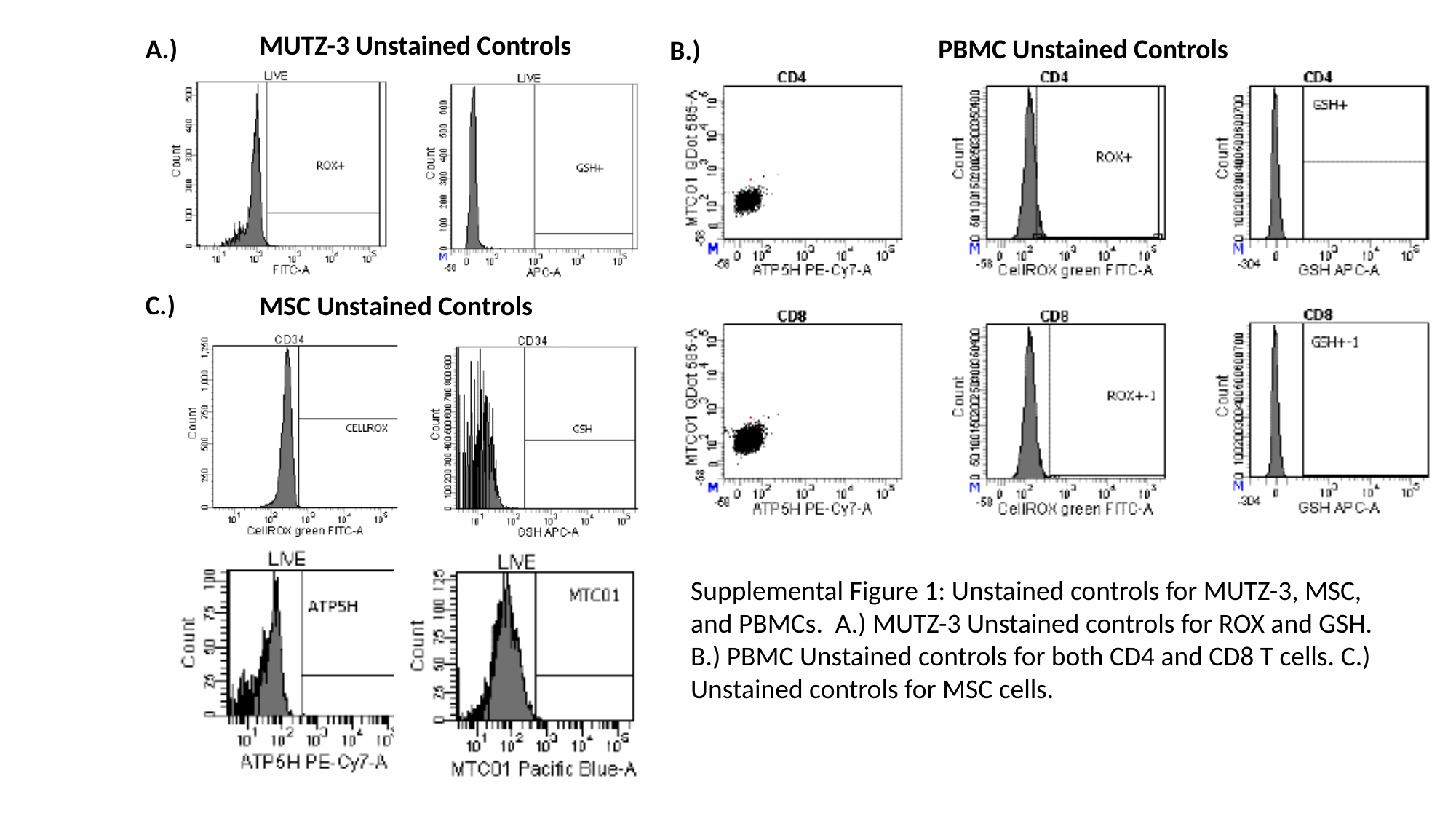

MUTZ-3 Unstained Controls
A.)
PBMC Unstained Controls
B.)
C.)
MSC Unstained Controls
Supplemental Figure 1: Unstained controls for MUTZ-3, MSC, and PBMCs. A.) MUTZ-3 Unstained controls for ROX and GSH. B.) PBMC Unstained controls for both CD4 and CD8 T cells. C.) Unstained controls for MSC cells.
