# Supplementary material for: A Novel FACS-Based Workflow for Simultaneous Assessment of RedOx Status, Cellular Phenotype, and Mitochondrial Genome Stability
Source: Biochem (Basel). Author manuscript; Available in PMC 2022 Aug 5. (PMC9355044; doi:10.3390/biochem1010001)
Supplement: Supplemental Material (Zip File) [file NIHMS1822005-supplement-Supplemental_Material__Zip_File_.zip › Supplemental_Figures_Biochem/Supplemental Figure 2.pptx]

## Slide 1
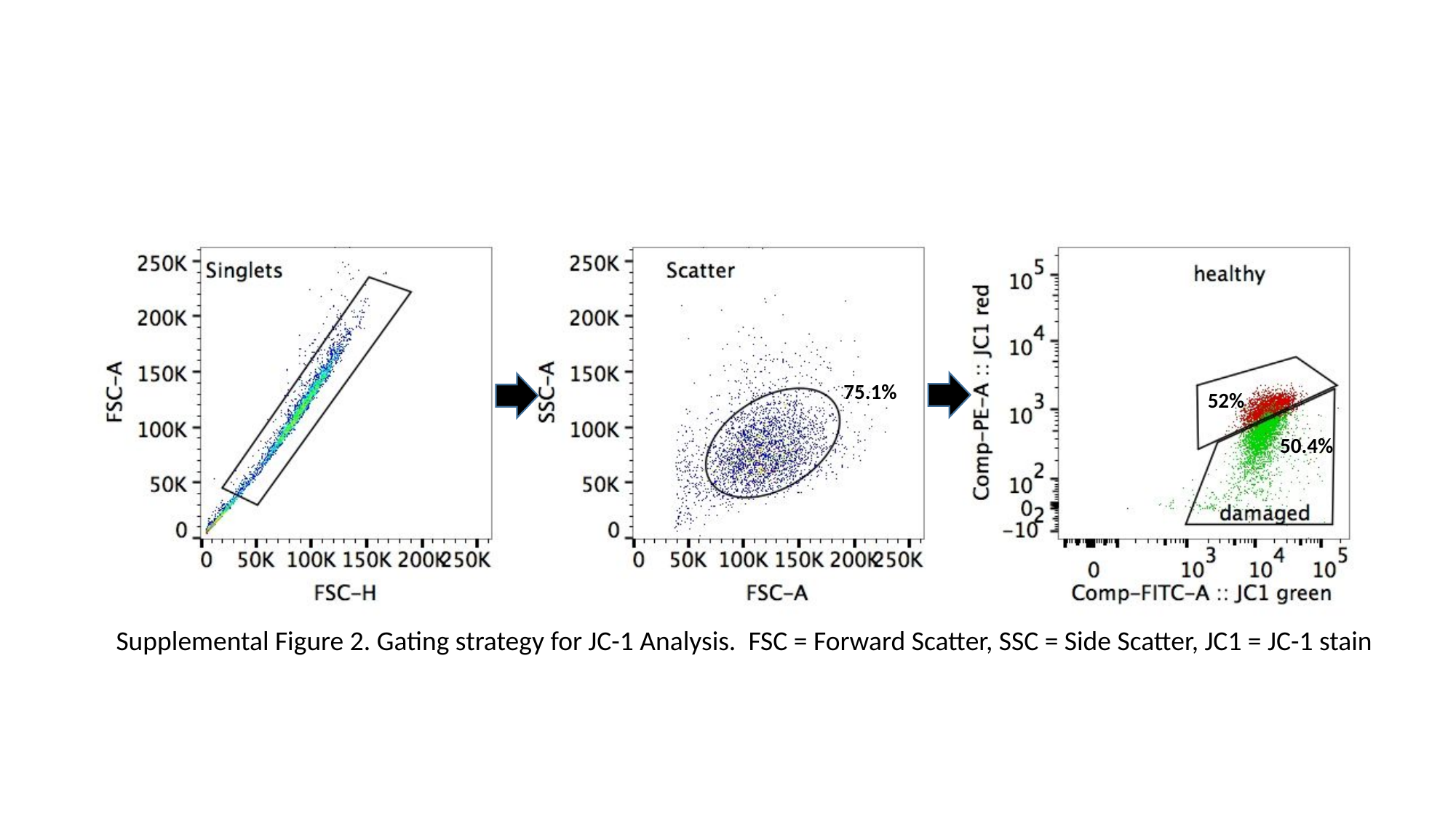

75.1%
52%
50.4%
Supplemental Figure 2. Gating strategy for JC-1 Analysis. FSC = Forward Scatter, SSC = Side Scatter, JC1 = JC-1 stain
