# Supplementary material for: A Novel FACS-Based Workflow for Simultaneous Assessment of RedOx Status, Cellular Phenotype, and Mitochondrial Genome Stability
Source: Biochem (Basel). Author manuscript; Available in PMC 2022 Aug 5. (PMC9355044; doi:10.3390/biochem1010001)
Supplement: Supplemental Material (Zip File) [file NIHMS1822005-supplement-Supplemental_Material__Zip_File_.zip › Supplemental_Figures_Biochem/Supplemental Figure 3.pptx]

## Slide 1
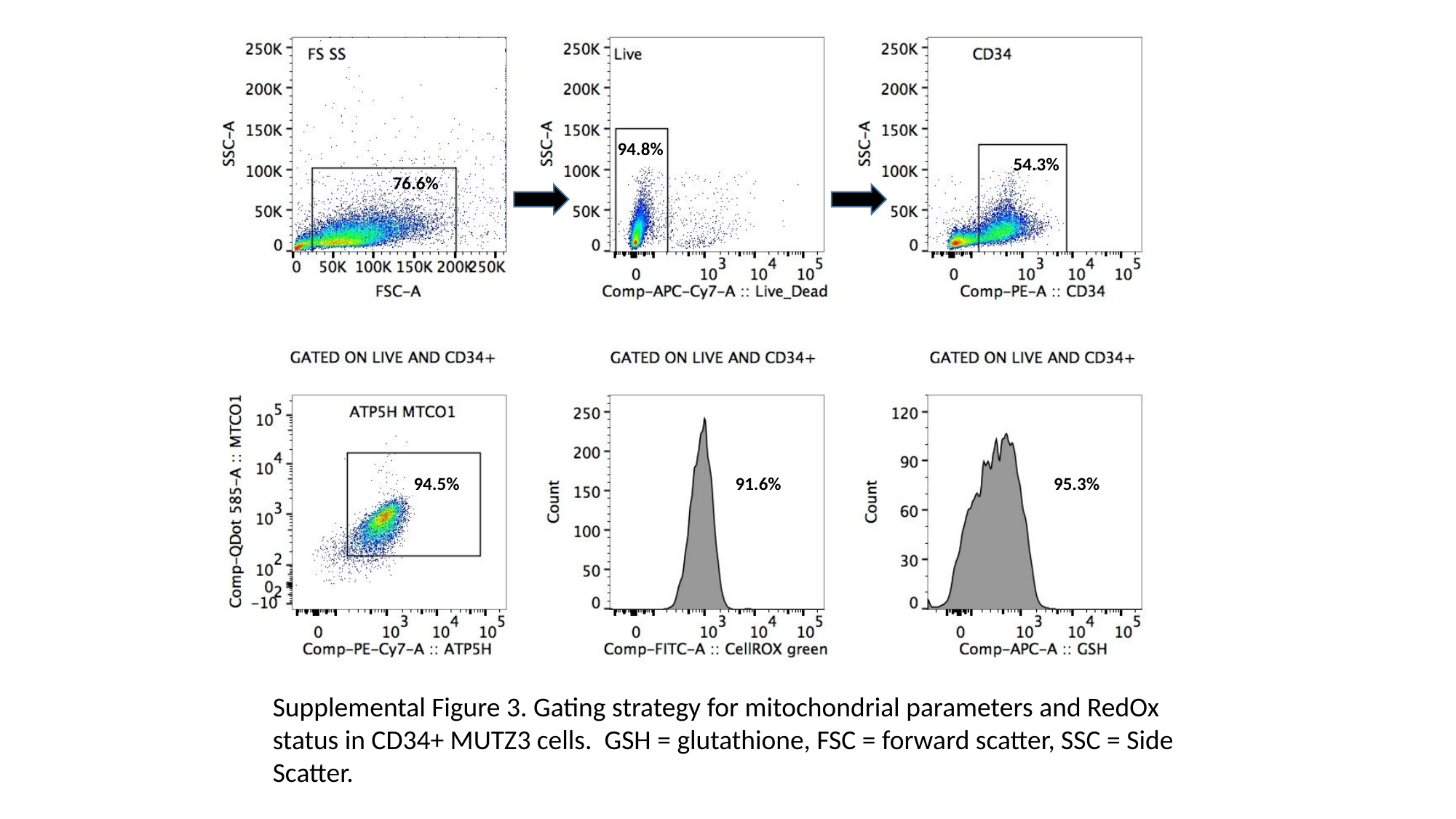

94.8%
54.3%
76.6%
95.3%
94.5%
91.6%
Supplemental Figure 3. Gating strategy for mitochondrial parameters and RedOx status in CD34+ MUTZ3 cells. GSH = glutathione, FSC = forward scatter, SSC = Side Scatter.
