# Supplementary material for: A Novel FACS-Based Workflow for Simultaneous Assessment of RedOx Status, Cellular Phenotype, and Mitochondrial Genome Stability
Source: Biochem (Basel). Author manuscript; Available in PMC 2022 Aug 5. (PMC9355044; doi:10.3390/biochem1010001)
Supplement: Supplemental Material (Zip File) [file NIHMS1822005-supplement-Supplemental_Material__Zip_File_.zip › Supplemental_Figures_Biochem/Supplemental Figure 4.pptx]

## Slide 1
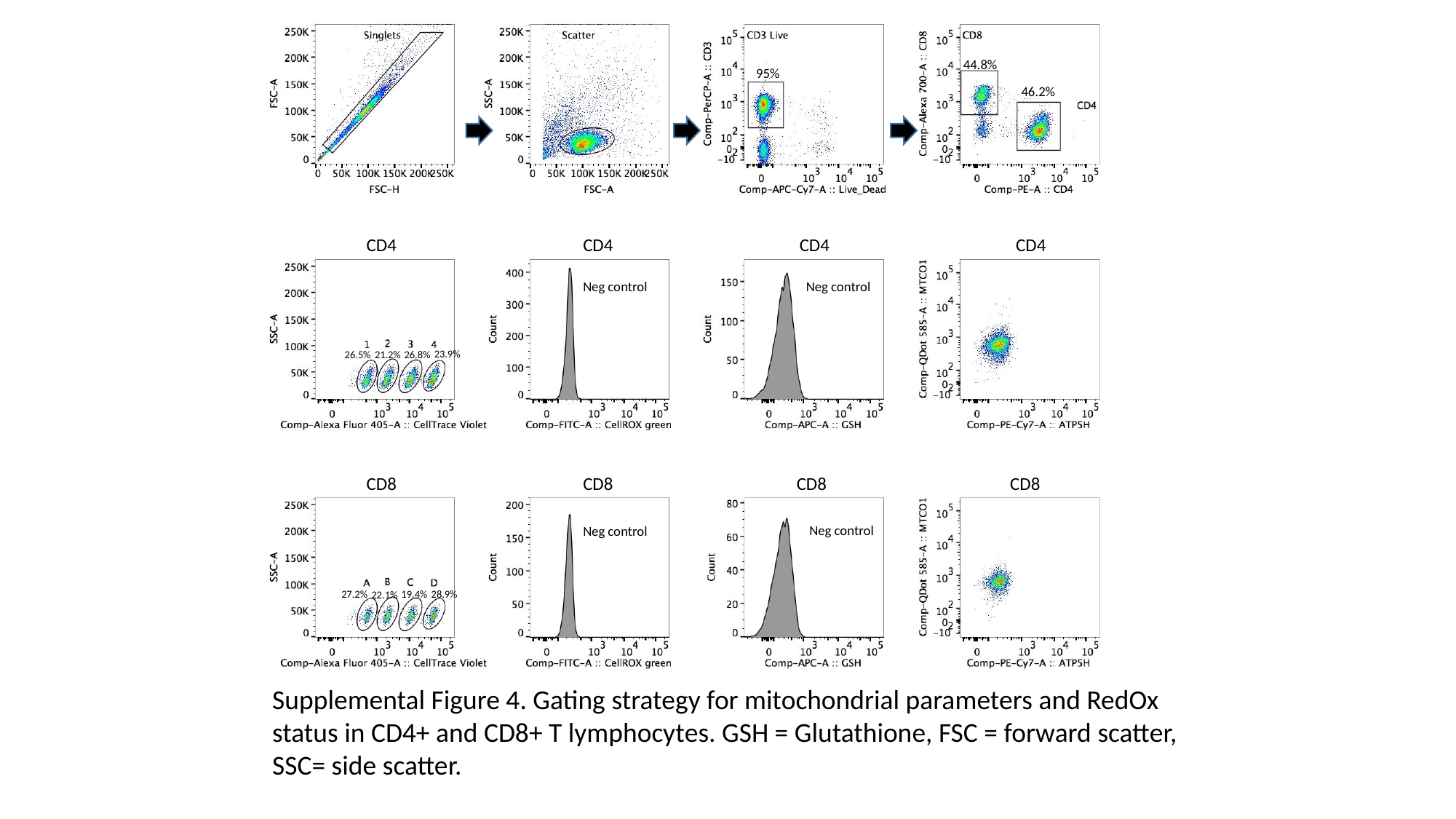

44.8%
95%
46.2%
CD4
CD4
CD4
CD4
Neg control
Neg control
23.9%
26.8%
26.5%
21.2%
CD8
CD8
CD8
CD8
Neg control
Neg control
28.9%
19.4%
27.2%
22.1%
Supplemental Figure 4. Gating strategy for mitochondrial parameters and RedOx status in CD4+ and CD8+ T lymphocytes. GSH = Glutathione, FSC = forward scatter, SSC= side scatter.
