# Supplementary material for: A Novel FACS-Based Workflow for Simultaneous Assessment of RedOx Status, Cellular Phenotype, and Mitochondrial Genome Stability
Source: Biochem (Basel). Author manuscript; Available in PMC 2022 Aug 5. (PMC9355044; doi:10.3390/biochem1010001)
Supplement: Supplemental Material (Zip File) [file NIHMS1822005-supplement-Supplemental_Material__Zip_File_.zip › Supplemental_Figures_Biochem/Supplemental Figure 5.pptx]

## Slide 1
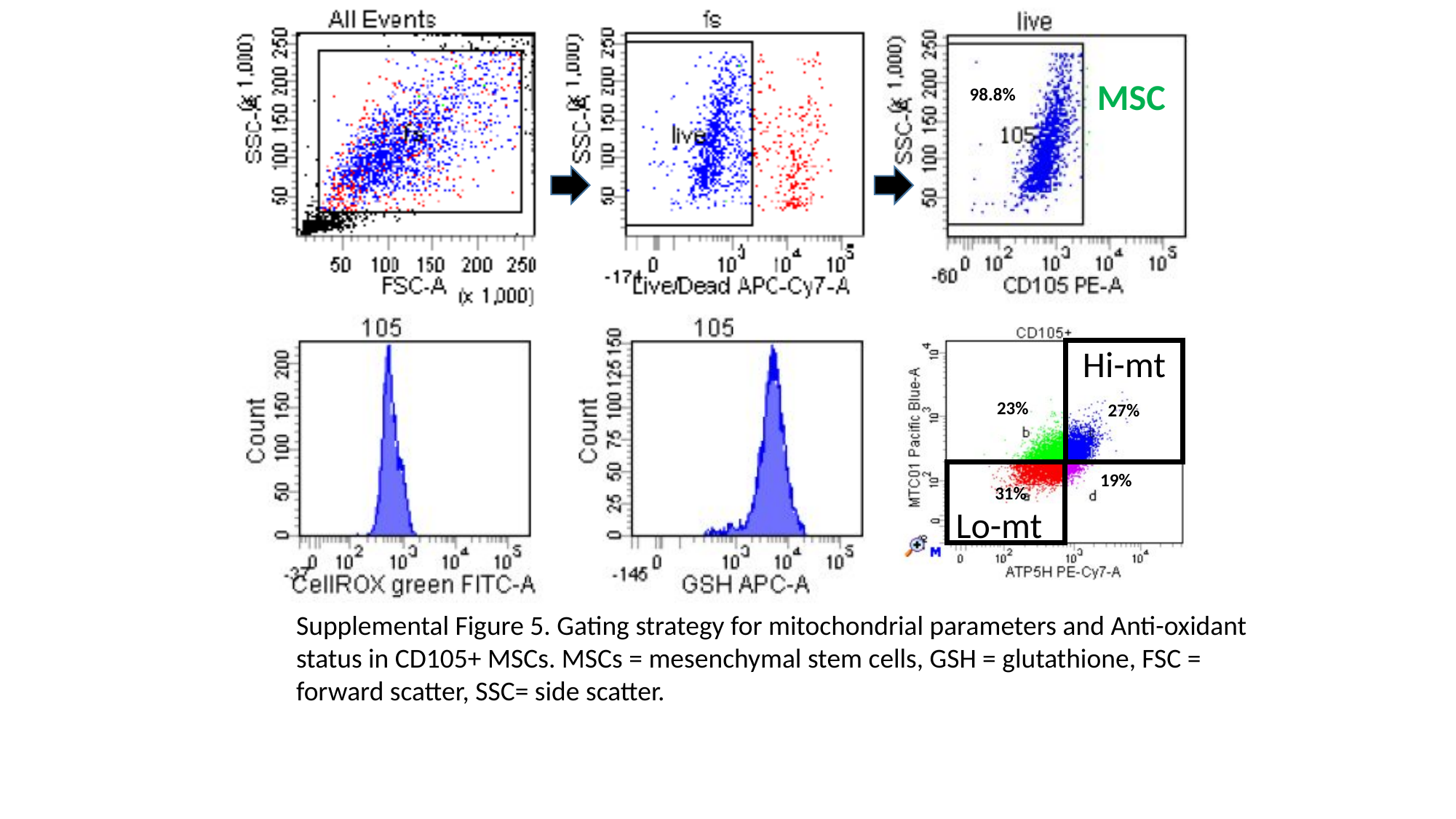

MSC
Hi-mt
Lo-mt
98.8%
23%
27%
19%
31%
Supplemental Figure 5. Gating strategy for mitochondrial parameters and Anti-oxidant status in CD105+ MSCs. MSCs = mesenchymal stem cells, GSH = glutathione, FSC = forward scatter, SSC= side scatter.
